# Accelerated Biological Aging Increases the Risk of Head and Neck Cancer: Insights From Genetic Instruments of Epigenetic Clocks

**DOI:** 10.1002/mc.70144

**Published:** 2026-07-02

**Authors:** Jiaqi Wang, Jie Deng, Yifan Xu, Chia‐Wen Tsai, Wen‐Shin Chang, Yang Deng, Maosheng Huang, Da‐Tian Bau, Guojun Li, Jian Gu

**Affiliations:** ^1^ Department of Epidemiology The University of Texas MD Anderson Cancer Center Houston Texas USA; ^2^ Department of General Surgery, Yangpu Hospital Tongji University School of Medicine Shanghai People's Republic of China; ^3^ School of Public Health The University of Texas Health Science Center at Houston Houston Texas USA; ^4^ Terry Fox Cancer Research Laboratory China Medical University Hospital Taichung Taiwan; ^5^ Department of Head and Neck Surgery The University of Texas MD Anderson Cancer Center Houston Texas USA

**Keywords:** biological aging, epigenetic clock, head and neck cancer, Mendelian randomization, PGS, SNP

## Abstract

Epigenetic clocks are robust biomarkers of biological aging and have been associated with cancer susceptibility. However, the relationship between genetically predicted epigenetic age acceleration and head and neck cancer risk remains unclear. Using a large case‐control study of 2189 head and neck squamous cell carcinoma (HNSCC) cases and 2189 age‐ and sex‐matched controls, we investigated the associations between polygenic scores (PGSs) for multiple epigenetic clocks and HNSCC risk, and evaluated their potential causal roles using two‐sample Mendelian randomization (MR). Genome‐wide association study (GWAS)‐identified single nucleotide polymorphisms (SNPs) associated with four epigenetic clocks (HannumAge, HorvathAge, GrimAge, and PhenoAge) were used to construct clock‐specific PGSs. Logistic regression models were applied to assess associations between PGSs and HNSCC risk, while MR analyses, including inverse‐variance weighted (IVW), weighted median, and MR‐Egger methods, were used to infer potential causal relationships. Among the 48 epigenetic clock‐associated SNPs, 12 showed nominal associations with HNSCC risk, and one variant (rs2275558 in *PBX1*) remained significant after Bonferroni correction (OR = 0.67, 95% CI: 0.60–0.76). PGSs for all four epigenetic clocks were higher in cases than in controls. In logistic regression analyses, each standard deviation increase in HannumAge PGS was associated with a 25% higher risk of HNSCC (OR = 1.25, 95% CI: 1.10–1.41), whereas HorvathAge, GrimAge, and PhenoAge PGSs showed weaker positive associations (ORs ranging from 1.06 to 1.10). Individuals in the highest PGS quartile for all four epigenetic clocks exhibiting 14%–25% higher risk than those in the lower three quartiles. MR analyses supported potential causal effects of genetically predicted HannumAge (IVW OR = 1.24 per SD increase, 95% CI: 1.09–1.42) and GrimAge (IVW OR = 1.23 per SD increase, 95% CI: 0.98–1.56) on HNSCC risk, with consistent estimates in weighted median analyses. Our results highlight biological aging as a potential etiologic mechanism for HNSCC and suggest that epigenetic clock‐related genetic profiles may improve HNSCC risk stratification.

**Abbreviations**
GWAS: Genome‐wide association study
HNC: Head and neck cancer
HNSCC: Head and neck squamous cell carcinoma
IVW: Inverse‐variance weighting
MDACC: MD Anderson cancer center
MR: Mendelian randomization
PGS: Polygenic score
SNP: Single nucleotide polymorphism

## 1 Introduction

Head and neck cancer (HNC) refers to a group of diverse malignancies arising in the upper aerodigestive tract, including oral cavity, pharynx, larynx, nasal cavity, and salivary glands [1]. HNC is the 7th most common cancer worldwide with close to one million new cases annually [2]. Additionally, HNC leads to nearly half a million deaths globally [2]. In the United States, HNC is the 9th most diagnosed cancer with estimated 77,770 new cases and 17,110 new deaths in 2026 [3]. The overall incidences of HNC have been steadily increasing in the past three decades both globally and in the United States [4, 5, 6, 7]. Globally, the increasing incidence of HNC is largely due to increased tobacco smoking, alcohol drinking, and betel quid chewing [5]. Tobacco smoking and alcohol drinking are two major risk factors for HNC, and they exhibit a strong synergistic interaction, with heavy consumption of both increasing HNC risk by 35‐fold [8]. In the United States, the increasing incidence of overall HNC is driven primarily by the dramatic increase in oropharyngeal cancer, which are mainly caused by infection with high‐risk human papillomavirus (HPV) [9], whereas HNC in other anatomic sites that are more strongly linked to smoking and drinking, such as oral cavity, larynx, and hypopharynx, have decreased due to reduced smoking rate [4, 5, 6, 7, 8].

Aging is one of the strongest determinants of cancer risk. Most HNC cases occur in older populations. The most recent Surveillance, Epidemiology, and End Results (SEER) program estimated that the 5‐year (2018–2022) age‐adjusted incidence rates of HNC were 60.5, 34.6, and 2.6 per 100,000 people for those aged 65 and older, 50–64 years, and younger than 50 years, respectively [10]. The median age at diagnosis for HNC is 65 and over 70% of new HNC patients are 55 years and older [11]. There are multiple contributing factors for the increased cancer risks among older populations, including increased carcinogen exposure, decreased essential cellular functions such as DNA repair, accumulation of genetic mutations and epigenetic aberrations, and diminished immune surveillance [12, 13].

Although aging is the dominant risk factor for most cancers, risk varies markedly among individuals of the same age. Biological age, derived from clinical and molecular markers of aging, more accurately reflects the physiological decline associated with advanced age than chronological age itself. Biological age serves as a biomarker of chronological age and provides additional discriminatory power for risk stratification of aging‐related diseases. Multiple biological age estimators have been developed, such as clinical indices, telomere length, omics‐based models, image‐based models, and composite biomarkers [14, 15, 16, 17, 18, 19]. Accelerated aging, defined as biological age exceeding chronological age, is associated with increased morbidity and mortality [19, 20, 21, 22].

Among all the biological age estimators, epigenetic clocks are widely considered to be the most accurate [23, 24]. Numerous epigenetic clocks have been developed based on distinct sets of CpG sites across various tissues, among which HannumAge, HorvathAge, PhenoAge, and GrimAge are four of the most extensively studied for their associations with disease risk and mortality [19, 25, 26, 27, 28]. In addition, genetic predictors of these epigenetic clocks have been identified through genome‐wide association studies (GWAS), and an increasing number of studies have used these genetic instruments as proxies to evaluate the associations between epigenetic clocks and disease risk [21, 22, 29, 30, 31, 32, 33, 34, 35]. To date, no study has reported the associations between epigenetic clocks and the risk of HNC. In this study, using a large case‐control design, we evaluated, for the first time, polygenic scores (PGSs) for four epigenetic clocks as potential risk factors for HNC and applied Mendelian randomization (MR) analysis to investigate the associations between these epigenetic clocks and HNC risk.

## 2 Methods

### 2.1 Study Population

This is a retrospective case control study consisting of 2189 patients diagnosed with head and neck squamous cell carcinoma (HNSCC) and an equal number of cancer‐free controls. HNSCC is the predominant histology and accounts for about 90% of all HNC. All cases were newly diagnosed, histologically confirmed HNSCC patients recruited from the University of Texas MD Anderson Cancer Center (MDACC). The recruitment strategy has been described previously [36, 37, 38]. No restrictions on age, sex, or ethnicity were applied during case enrollment. The controls were recruited from genetically unrelated companions of cancer patients at MDACC or from individuals previously enrolled in an MDACC bladder cancer case control study [39]. Controls were matched to cases by age (within 1 year) and sex.

### 2.2 Genotyping, Imputation, and SNP Proxies for Epigenetic Clocks

The genotyping platforms, analysis pipeline, quality control, and imputation procedures have been described previously [36, 39]. Briefly, genotyping was performed using Illumina HumanHap610 or HumanOmniExpress‐12v1 BeadChip following the manufacturer's protocols. Genotype imputation was conducted using the Michigan Imputation Server (imputationserver.sph.umich.edu) with 1000 Genomes Project data as the reference panel. The SNP proxies for the four epigenetic clocks were selected based on previous GWAS [32, 33]. Specifically, 9 SNPs were selected for HannumAge, 24 for HorvathAge, 11 for PhenoAge, and 4 for GrimAge. These SNPs were used to construct PGS for the respective epigenetic clocks and as genetic instruments for summary‐statistics based MR analyses.

### 2.3 Statistical Analysis

The statistical analyses were performed as previously described [33, 34]. Briefly, continuous variables, including age and PGS, were compared between cases and controls using Student's *t*‐test, whereas categorical variables, including genotype frequency and dichotomized PGS, were evaluated using the *χ* ^2^ test or Fisher's exact test, as appropriate. PGS for each epigenetic clock was generated based on the corresponding SNPs following published methods [40, 41]. Multivariable logistic regression was used to assess the associations of each SNP and PGS with HNSCC risk adjusting for age. MR analyses were performed using the inverse‐variance weighted (IVW) approach, with MR‐Egger regression and the weighted median method applied as sensitivity analyses. Expression quantitative trait loci (eQTL) analysis was performed using the GTEx portal website (https://gtexportal.org). All statistical analyses were conducted in R package, and all *p* values were two‐sided with statistical significance defined as *p* < 0.05.

## 3 Results

### 3.1 Characteristics of the Study Population

The study population comprised 2189 HNSCC cases and an equal number of controls (Table 1). Controls were individually matched to cases by age (within 1 year) and sex. The mean age at diagnosis among cases was 57.91 ± 11.20 years, compared with 58.41 ± 10.34 years at enrollment among controls. Consistent with the known epidemiology of HNSCC, men comprised the majority of cases (77%).

**Table 1:** Main characteristics of the cases and controls.

Variables	Cases, *N* (%)	Controls, *N* (%)		*p* value
Age, mean (SD)	57.91 (11.20)	58.41 (10.34)		0.125
Sex				
Male	1685 (76.98)	1685 (76.98)		
Female	504 (23.02)	504 (23.02)		1.000

### 3.2 Associations of PGSs of Epigenetic Clocks With HNSCC Risk

First, 48 SNPs previously identified through GWAS as instrumental variants for different epigenetic clocks were evaluated for their associations with HNSCC risk (Table 2). Of these, 12 variants showed nominal associations with HNSCC susceptibility (*p* < 0.05), only one (rs2275558 in PBX1, OR = 0.67, 95% CI: 0.6–0.76) remained significant after Bonferroni correction (*p* < 0.001).

**Table 2:** Individual associations of epigenetic clocks‐associated SNPs with HNSCC risk.

SNP ID	Chr	Position	Gene	Allele	*β*	EAF		OR (95% CI)	*p*‐value
						Cases	Controls		
HannumAge									
rs34970912	16	73068163	ZFHX3	G/C	0.521	0.035	0.025	1.39 (1.09–1.79)	**0.009**
rs12417758	11	66076360	RP11‐867G23.13	C/T	0.209	0.451	0.425	1.11 (1.02–1.21)	**0.012**
rs1005277	10	38218259	ZNF25	A/C	0.301	0.289	0.272	1.08 (0.99–1.19)	0.089
rs3093956	6	31426967	—	C/T	0.243	0.155	0.145	1.08 (0.96–1.21)	0.217
rs10786282	10	98122808	OPALIN	G/A	0.360	0.795	0.786	1.05 (0.95–1.17)	0.317
rs4383328	2	16693124	AC104623.2	T/A	0.189	0.734	0.725	1.04 (0.95–1.15)	0.377
rs111731678	7	130418744	KLF14	T/A	0.227	0.797	0.803	0.95 (0.86–1.06)	0.382
rs1598856	4	103446115	NFKB1	A/G	0.186	0.456	0.448	1.03 (0.95–1.12)	0.453
rs4838595	10	49675247	ARHGAP22	C/T	0.258	0.877	0.882	0.96 (0.84–1.09)	0.530
HorvathAge									
rs2275558	1	164529120	PBX1	G/A	0.234	0.808	0.830	0.67 (0.6–0.76)	**< 0.001**
rs75243280	22	17601466	CECR6	C/T	0.232	0.323	0.297	1.17 (1.06–1.29)	**0.001**
rs12666349	7	31728180	PPP1R17	T/C	0.255	0.816	0.833	0.85 (0.76–0.95)	**0.005**
rs3917672	1	169592981	SELP	G/A	0.262	0.511	0.536	0.9 (0.83–0.98)	**0.017**
rs1488106	3	168859006	MECOM	T/C	0.183	0.377	0.352	1.11 (1.02–1.21)	**0.020**
rs1726672	1	236519502	EDARADD	C/T	0.204	0.702	0.681	1.1 (1.01–1.21)	**0.037**
rs34003787	16	73071381	ZFHX3	T/C	0.324	0.089	0.078	1.15 (0.99–1.34)	0.072
rs144317085	4	105806108	RP11‐556I14.2	A/T	0.514	0.965	0.971	0.83 (0.65–1.05)	0.117
rs79111787	3	47715545	SMARCC1	C/T	0.908	0.014	0.011	1.29 (0.88–1.88)	0.190
rs57941717	21	38374179	RIPPLY3	T/G	0.291	0.247	0.237	1.06 (0.96–1.17)	0.242
rs1511762	18	42119324	LINC01478	T/C	0.264	0.232	0.222	1.06 (0.96–1.17)	0.248
rs12903325	15	50353277	ATP8B4	G/T	0.222	0.223	0.232	0.95 (0.86–1.05)	0.361
rs4240228	2	16688759	AC104623.2	T/G	0.255	0.734	0.725	1.04 (0.95–1.15)	0.377
rs2736099	5	1287340	TERT	A/G	0.233	0.357	0.350	1.04 (0.95–1.14)	0.402
rs6577536	1	8910110	ENO1	A/G	0.197	0.485	0.477	1.04 (0.95–1.13)	0.403
rs10735418	12	107343376	RP11‐412D9.4	T/C	0.195	0.612	0.605	1.03 (0.95–1.12)	0.478
rs6414374	3	150001224	LINC01214	A/G	0.321	0.160	0.156	1.04 (0.93–1.17)	0.500
rs7627756	3	160217483	KPNA4	A/G	0.216	0.553	0.560	0.98 (0.9–1.06)	0.581
rs2492286	3	128336298	RPN1	T/G	0.281	0.156	0.152	1.03 (0.92–1.16)	0.636
rs10447389	6	25642577	ZFP57	G/A	0.276	0.718	0.721	0.98 (0.89–1.08)	0.702
rs10949481	6	18121029	NHLRC1	A/T	1.082	0.953	0.954	0.98 (0.8–1.19)	0.807
rs12043492	1	39457006	AKIRIN1	T/C	0.217	0.420	0.417	1.01 (0.93–1.1)	0.823
rs7550821	1	208029947	C1orf132	C/T	0.255	0.765	0.767	0.99 (0.9–1.09)	0.833
rs10732882	11	57111693	P2RX3	G/T	0.241	0.603	0.602	1 (0.92–1.09)	0.978
GrimAge									
rs887466	6	31143511	POU5F1	G/A	0.193	0.634	0.607	1.12 (1.03–1.22)	**0.010**
rs4065321	17	38143548	PSMD3	C/T	0.170	0.465	0.449	1.06 (0.98–1.16)	0.150
rs9386796	6	109618704	CCDC162P	T/C	0.198	0.434	0.446	0.95 (0.87–1.03)	0.241
rs17094148	10	101280279	LINC01475	G/A	0.180	0.290	0.282	1.04 (0.95–1.14)	0.433
PhenoAge									
rs7228835	18	41969071	LINC01478	G/C	0.514	0.883	0.902	0.82 (0.72–0.94)	**0.004**
rs678553	1	236525447	EDARADD	T/C	0.326	0.697	0.675	1.11 (1.01–1.21)	**0.030**
rs752223	1	60433076	RN7SL475P	G/A	0.560	0.920	0.927	0.91 (0.78–1.07)	0.260
rs73028070	11	122681835	UBASH3B	G/A	0.433	0.920	0.924	0.93 (0.79–1.09)	0.349
rs11190127	10	101271982	LINC01475	A/C	0.248	0.374	0.365	1.04 (0.95–1.14)	0.375
rs116853700	17	55466295	MSI2	A/G	0.552	0.042	0.039	1.09 (0.88–1.35)	0.418
rs6531114	2	16617781	AC010880.1	C/T	0.254	0.746	0.744	1.02 (0.93–1.13)	0.642
rs11253338	10	759559	DIP2C	T/C	0.285	0.183	0.179	1.02 (0.92–1.14)	0.676
rs1142345	6	18130918	TPMT	T/C	0.823	0.955	0.955	0.98 (0.8–1.21)	0.883
rs1990053	7	44925896	PURB	A/G	0.257	0.436	0.437	0.99 (0.91–1.08)	0.902
rs3829957	17	3378876	ASPA	C/T	0.380	0.812	0.811	1 (0.9–1.12)	0.938

*Note:* SNPs significantly associated with HNSCC risks (*p* < 0.05) are shown in bold.

**Figure 1:**
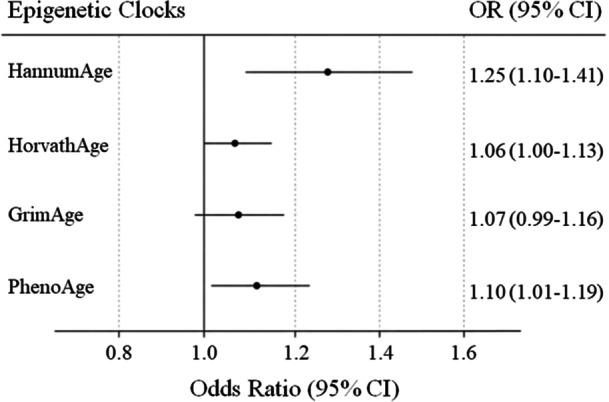
Associations of epigenetic clock‐specific polygenic scores (PGSs) with HNSCC risk. Odds ratios (ORs) and 95% confidence intervals (CIs) are shown per standard deviation (SD) increase in each PGS.

We next constructed PGSs for each of the four epigenetic clocks based on their corresponding proxy SNPs and compared the distributions between cases and controls (Table 3). The F‐statistics for the PGSs of HannumAge, HorvathAge, PhenoAge, and GrimAge were 18.1, 25.6, 8.0, and 4.3, respectively, suggesting strong genetic instruments for HannumAge and HorvathAge but weaker instruments for PhenoAge and GrimAge. PGSs were higher in cases than in controls for all four epigenetic clocks. Three of these differences reached statistical significance, including HannumAge (*p* = 0.0004), HorvathAge (*p* = 0.047), and PhenoAge (*p* = 0.021), whereas GrimAge showed a borderline significant difference (*p* = 0.082). In multivariable logistic regression analyses adjusted for age, each standard deviation increase in the HannumAge PGS was associated with a 25% higher risk of HNSCC (OR = 1.25, 95% CI: 1.10–1.41). The PGSs for HorvathAge, GrimAge, and PhenoAge also showed modest positive associations with HNSCC risk, with ORs (95% CIs) of 1.06 (1.00–1.13), 1.07 (0.99–1.16), and 1.10 (1.01–1.19), respectively (Figure 1).

**Table 3:** Comparisons of polygenic risk score (PRS) between cases and controls.

Epigenetic Clocks	Number of SNPs	PRS, Mean (SD)		*p* value
		Controls	Cases	
HannumAge	9	2.29 (0.47)	2.34 (0.50)	0.0004
HorvathAge	24	8.23 (0.93)	8.29 (1.02)	0.047
GrimAge	4	1.41 (0.74)	1.45 (0.74)	0.082
PhenoAge	11	5.59 (0.72)	5.64 (0.74)	0.021

To further evaluate risk stratification, PGSs were categorized using the median and the 75th percentile of the control distribution (Table 4). Individuals with a HannumAge PGS above the median had a 15% higher risk of HNSCC compared with those below the median (OR = 1.15, 95% CI: 1.02–1.29). Similarly, participants in the highest quartile of HannumAge PGS had a 25% increased risk relative to those in the lower three quartiles (OR = 1.25, 95% CI: 1.04–1.43).

**Table 4:** Associations of categorical polygenetic risk score (PRS) with HNSCC risk.

PRS	Control, *N* (%)	Case, *N* (%)	OR^a^ (95% CI)	*p* value
HannumAge				
Dichotomize at 50%				
Low	1095 (51.8)	1020 (48.2)	1 (reference)	
High	1094 (48.3)	1169 (51.7)	1.15 (1.02–1.29)	0.024
Dichotomize at 75%				
Low	1642 (51.5)	1545 (48.5)	1 (reference)	
High	547 (45.9)	644 (54.1)	1.25 (1.09–1.43)	0.001
HorvathAge				
Dichotomize at 50%				
Low	1095 (51.8)	1018 (48.2)	1 (reference)	
High	1094 (48.30	1171 (51.7)	1.15 (1.02–1.30)	0.019
Dichotomize at 75%				
Low	1642 (51.3)	1556 (48.7)	1 (reference)	
High	547 (46.4)	633 (53.6)	1.22 (1.07–1.40)	0.004
GrimAge				
Dichotomize at 50%				
Low	1138 (50.8)	1100 (49.2)	1 (reference)	
High	1051 (49.1)	1089 (50.9)	1.07 (0.95–1.21)	0.245
Dichotomize at 75%				
Low	1652 (50.8)	1599 (49.2)	1 (reference)	
High	537 (47.6)	590 (52.4)	1.14 (0.99–1.30)	0.066
PhenoAge				
Dichotomize at 50%				
Low	1095 (50.9)	1055 (49.1)	1 (reference)	
High	1094 (49.1)	1134 (50.9)	1.07 (0.95–1.21)	0.238
Dichotomize at 75%				
Low	1642 (51.2)	1568 (48.8)	1 (reference)	
High	547 (46.8)	621 (53.2)	1.19 (1.04–1.36)	0.013

a Multivariable logistic regression analysis adjusted by age.

For HorvathAge, GrimAge, and PhenoAge, participants with PGSs above the median also showed elevated HNSCC risk compared with those below the median. When comparing the highest quartile with the remaining participants, the corresponding increases in HNSCC risk were 22% for HorvathAge (OR = 1.22, 95% CI: 1.07–1.40), 14% for GrimAge (OR = 1.14, 95% CI: 0.99–1.30), and 19% for PhenoAge (OR = 1.19, 95% CI: 1.04–1.36), respectively.

### 3.3 Mendelian Randomization Analysis

To evaluate potential causal relationships, summary‐level MR analyses were conducted using the IVW method, with the weighted median and MR‐Egger regression approaches performed as sensitivity analyses (Table 5). Consistent with the PGS results, genetically predicted HannumAge acceleration was significantly associated with an increased risk of HNSCC (OR = 1.24 per SD increase, 95% CI: 1.09–1.42, *p* = 0.002). GrimAge also showed a borderline positive association (OR = 1.23, 95% CI: 0.98–1.56, *p* = 0.08). In contrast, no significant associations were observed for HorvathAge or PhenoAge in the MR analyses.

**Table 5:** Mendelian randomization analyses of epigenetic clocks and HNSCC risk.

Epigenetic Clocks	IVW		MR‐Egger		Weighted median	
	OR* (95% CI)	*p* value	OR* (95% CI)	*p* value	OR^a^ (95% CI)	*p* value
HannumAge	1.24 (1.09–1.42)	0.002	1.46 (0.79–2.72)	0.230	1.24 (1.04–1.49)	0.018
HorvathAge	1.02 (0.95–1.10)	0.598	0.95 (0.63–1.43)	0.813	1.03 (0.92–1.15)	0.653
GrimAge	1.23 (0.98–1.56)	0.080	0.13 (0.00–145.88)	0.571	1.32 (0.97–1.80)	0.078
PhenoAge	0.99 (0.90–1.08)	0.749	0.77 (0.56–1.06)	0.113	0.99 (0.87–1.13)	0.900

a OR per SD increase adjusted by age.

The weighted median analyses, which provide valid MR estimates if at least 50% of the SNPs in the genetic instrument are valid [42], yielded effect estimates comparable to those from the IVW analysis across all four epigenetic clocks. For HannumAge and GrimAge, the corresponding ORs per SD increase were 1.24 (95% CI: 1.04–1.49) and 1.32 (95% CI: 0.97–1.80), respectively. The MR‐Egger regression analysis produced a similar directional estimate for HannumAge (OR = 1.46, 95% CI: 0.79–2.72), although the association did not reach statistical significance (*p* = 0.23). For GrimAge, the MR‐Egger estimate differed from the IVW result and was accompanied by a wide confidence interval, suggesting limited statistical power.

## 4 Discussion

In this study, we investigated the associations between genetic predisposition to accelerated epigenetic aging and HNSCC risk using PGS and MR analyses. We found that higher PGSs for all four epigenetic clocks were associated with increased HNSCC risk, with individuals in the highest PGS quartile exhibiting 14%–25% higher risk than those in the lower three quartiles. MR analyses further supported potential causal effects of HannumAge and, to a lesser extent, GrimAge on HNSCC susceptibility.

Aging is one of the strongest established risk factors for HNSCC and biological aging provides a more informative measure of cancer susceptibility than chronological age alone. Epigenetic clocks have emerged as the most robust indicators of biological age. Accumulating evidence has linked epigenetic age acceleration to adverse health outcomes, including cancer incidence and mortality [19, 20, 21, 22, 25, 26, 27, 28, 43]. Recent MR studies have also evaluated genetic proxies of epigenetic clocks in relation to risks of breast, prostate, lung, colorectal, ovarian, and bladder cancers [33, 34, 35]. Compared with conventional observational studies, MR analyses are less susceptible to reverse causation and measurement variability that often complicate studies of intermediate phenotypes [44, 45, 46, 47]. Previous MR studies reported associations of GrimAge with colorectal cancer risk [33, 35], and of HannumAge and HorvathAge with bladder cancer risk [34], whereas no significant associations were observed for prostate, breast, lung, or ovarian cancers [33]. In the present study, we observed positive associations between all four epigenetic clocks and HNSCC risk, although the magnitudes varied across clocks.

The heterogeneity across epigenetic clocks likely reflects differences in their development and biological underpinnings. HannumAge and HorvathAge were trained to predict chronological age [25, 26], whereas GrimAge incorporates smoking history and methylation surrogates for seven mortality‐associated circulating proteins [27], and PhenoAge integrates chronological age with nine clinical biomarkers of physiological decline [28]. HorvathAge was derived from methylation profiles across 51 tissues and cell types, whereas the other clocks were developed using whole‐blood methylation data. The clocks also differ substantially in the number and composition of CpG sites included, ranging from 71 CpGs in HannumAge to 1030 in GrimAge, with minimal overlap among clocks. Given these methodological and biological differences, heterogeneous associations across cancer types are not unexpected. However, the consistent associations observed for all four PGSs in our study underscore the potential importance of biological aging in HNSCC etiology.

**Figure 2:**
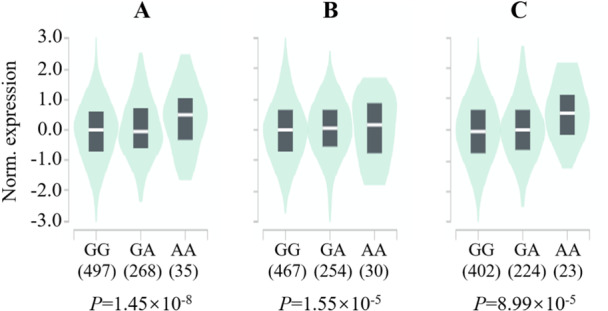
Effects of rs2275558 genotypes on *PBX1* mRNA expression based on expression quantitative trait loci (eQTL) analyses. The variant genotypes were significantly associated with increased *PBX1* mRNA expression in (A) whole blood, (B) sun‐exposed skin tissue from the lower leg, and (C) cultured fibroblasts. The *y*‐axis represents normalized PBX1 mRNA expression values, and the *x*‐axis indicates different genotypes and corresponding sample sizes. The violin plots and underlying data were obtained from the GTEx Portal.

In our PGS analyses based on individual‐level data, all four epigenetic clocks were associated with HNSCC risk, whereas summary statistics‐based MR analyses identified significant or borderline significant associations only for HannumAge and GrimAge. MR analysis relies on many assumptions [48], including three core assumptions: that the genetic instruments are strongly associated with the exposure, are independent of confounders, and influence disease risk exclusively through the exposure pathway. In this context, it is noteworthy that rs2275558 in *PBX1*, the only SNP remaining significant after Bonferroni correction, may affect HNSCC risk through mechanisms independent of biological aging. The variant allele was associated with higher HorvathAge acceleration (β = 0.234) but lower HNSCC risk (OR = 0.67, 95% CI: 0.60–0.76) (Table 2). When this SNP was excluded from the HorvathAge PGS, the association between HorvathAge and HNSCC became stronger (OR = 1.09, 95% CI: 1.03–1.16, *p* = 0.0055, data not shown) compared with the original model that included the SNP (OR = 1.06, 95% CI: 1.00–1.13, *p* = 0.05; Figure 1). PBX1 is a key transcription factor involved in multiple oncogenic pathways, including proliferation, apoptosis resistance, angiogenesis, epithelial–mesenchymal transition, metastasis, immune evasion, genome instability, and metabolic dysregulation [49]. In HNSCC, PBX1 has been suggested to function as a tumor suppressor, as its expression is significantly lower in tumors than in normal tissues and reduced expression is associated with poor prognosis [50]. rs2275558 is a missense variant (Gly21Ser) located in exon 1 of *PBX1*. Although the impact of this amino acid substitution on protein structure and function is not immediately apparent, eQTL analyses demonstrated that the variant genotypes were consistently and significantly associated with increased *PBX1* mRNA expression across multiple tissues and cells (Figure 2). The increased expression associated with the variant genotypes is consistent with the suggested tumor suppressor role of PBX1 and with the protective effect of the variant genotypes on HNSCC risk observed in our study.

In conclusion, in this large case control study, we observed significant associations for PGS of four epigenetic clocks with increased HNSCC risk, and MR analyses provide further evidence supporting biological aging as a potential etiologic mechanism for HNSCC and suggest that epigenetic clock‐related genetic profiles may improve HNSCC risk stratification.

## Author Contributions

J.G., D.‐T.B., G.L., J.W., and Y.D. conceived and designed the study. J.W., J.D., and M.H. performed the statistical analyses. Y.X., C.‐W.T., and W.‐S.C. contributed to data acquisition, data curation, and methodology development. J.G. and G.L. provided resources. J.G. supervised the study. J.W. and J.G. drafted the original manuscript. J.D., Y.X., C.‐W.T., W.‐S.C., Y.D., M.H., D.‐T.B., and G.L. critically reviewed and revised the manuscript.

## Ethics Statement

This study was approved by the Institutional Review Board of the University of Texas MD Anderson Cancer Center (Protocol # LAB00‐062 and LAB98‐040). Written informed consent was obtained from all participants.

## Conflicts of Interest

The authors declare no conflicts of interest.

## Data Availability

The data that support the findings of this study are available on request from the corresponding author. The data are not publicly available due to privacy or ethical restrictions.
